# Association Between Obesity and Sex-Related Survival Difference in Lung Cancer

**DOI:** 10.1200/CCI-25-00263

**Published:** 2026-04-02

**Authors:** Alexey Ryzhenkov, Salma Rachidi, Valtteri Nieminen, Marianna Niemi, Mads Albrecht Andersen, Johanna Niklander, Juuso Paajanen, Pooja Bhatnagar, Andreas Bjerrum, Paula Kauppi, Stelios Theophanous, Wei Hai Deng, Zarah Van Schoor, Annelies Verbiest, Åslaug Helland, Johanna Sanoja, Annelies Janssens, Aija Knuuttila, Ilkka Ilonen, Eric Fey, Kimmo Porkka

**Affiliations:** ^1^ Departments of Oncology and Hematology, iCAN Digital Precision Cancer Medicine Flagship, University of Helsinki, and Helsinki University Hospital Comprehensive Cancer Center, Helsinki, Finland; ^2^ HUS IT Management, Helsinki University Hospital, Helsinki, Finland; ^3^ Department of Computing, University of Turku, Turku, Finland; ^4^ Department of Oncology, Copenhagen University Hospital, Copenhagen, Denmark; ^5^ Heart and Lung Center, Pulmonary Department, Helsinki University Hospital, Helsinki, Finland; ^6^ Leeds Teaching Hospitals NHS Trust, Leeds, United Kingdom; ^7^ Division of Cancer Medicine, Oslo University Hospital, Oslo, Norway; ^8^ Department of Medical Oncology, Antwerp University Hospital, Antwerp, Belgium; ^9^ MIPRO, University of Antwerp, Antwerp, Belgium; ^10^ Department of Thoracic and Esophageal Surgery, Heart and Lung Center, Helsinki University Hospital and University of Helsinki, Helsinki, Finland

## Abstract

**PURPOSE:**

Survival discrepancy between male and female patients in lung cancer is a well-known, but still poorly understood phenomenon. Previous studies have used different patient cohorts and clinical covariates and have not included obesity, which is associated with longer lung cancer survival. We evaluated the relationship between survival, obesity, sex and other covariates using comprehensive, harmonized patient cohorts and a federated analysis approach.

**MATERIALS AND METHODS:**

Initial analyses were done in a retrospective, real-world cohort of 7,327 patients with lung cancer diagnosed at the Helsinki University Hospital from 2015 to 2024. Patients were stratified by BMI, and univariate and multivariate analyses of survival were performed. External validation of univariate analyses was performed on data from four European university hospitals (n = 12,700).

**RESULTS:**

Higher BMI was associated with a smaller sex-related survival difference. In the normal BMI cohort (18.5-25 kg/m^2^), the 2-year overall survival was 46% in females and 29% in males (*P* < .01). In the high BMI cohort, the difference was 51% versus 41% (*P* < .01). Similar trends were observed in the validation sites, with some variation. The largest effect of high BMI was observed in squamous cell carcinoma. When full multivariate analysis was performed separately for high and normal BMI patients, the effect of male sex on survival was 32% smaller among high BMI patients.

**CONCLUSION:**

Higher BMI was associated with reduced survival gap between sexes, emphasizing the value of comprehensive covariate reporting in future clinical trials and observational studies.

## INTRODUCTION

Lung cancer accounts for 19% of all cancer deaths, with an annual incidence of approximately 2.5 million cases.^1^ Better survival outcomes in female over male patients with lung cancer have been reported since 1990s.^2^ Multiple studies have shown that adjusting for clinical covariates, such as stage, treatment, and age, does not fully explain the association between sex and survival, suggesting that sex is an independent prognostic factor in lung cancer.^3-6^ However, other studies have not supported these findings, reporting that controlling for covariates eliminates or significantly reduces the survival difference between male and female patients.^7-9^

**CONTEXT**

**Key Objective**
What is the association between BMI and survival discrepancy of sexes in lung cancer within a novel Observational Medical Outcomes Partnership–based federated cohort across four cancer care sites in Europe?
**Knowledge Generated**
There is evidence of the association between BMI and sex-related survival discrepancy, while the effect size varies by histology and geographical location. There is also variance in sex ratios in different BMI groups among treatment locations.
**Relevance *(P.-M. Putora)***
This large real-world data analysis demonstrates the value of granular data, common data models, and external validation, providing valuable insights into clinically relevant issues. The result of the study demonstrates the importance of BMI as a relevant factor in lung cancer survival (significantly interacting with gender).*
*Relevance section written by *JCO CCI* Associate Editor Paul-Martin Putora, MD, PhD, MA.

It was further reported that the presence and magnitude of the survival difference between male and female patients depends on the analyzed cohort; for example, differences among patients with adenocarcinoma have been reported to be larger than in other histologic subgroups.^10^ Survival discrepancies may also depend on the geographical location because of different population characteristics and treatment practices.^11^

In addition to these commonly analyzed factors, obesity was shown to be associated with better survival^12-14^ in lung cancer. Because obesity is usually associated with negative effects on health, this phenomenon has been called the obesity paradox.^15,16^ Obesity rates are also increasing worldwide,^17^ urging for a better understanding of the specifics of this patient group. As obesity varies at least by sex, age, and geographical location,^18,19^ its total influence on survival could vary in different patient cohorts. Most of the previous studies on sex-associated survival differences have not included obesity or BMI as a covariate or stratification factor, nor have they reported the proportion of obese patients in their study populations.^2-11^ In addition, they used different clinical cohorts,^2,6,8^ geographic locations,^3,9,20^ and statistical methods,^3,5,9^ which limits the comparability of these studies. Finally, many studies on the topic^13,15,21^ have focused on non–small cell lung cancer (NSCLC) excluding other lung cancer types commonly present in clinical practice.

To overcome these limitations and describe the effect of obesity on sex-associated survival differences, we assembled a large retrospective cohort of patients with cancer from a single hospital in Finland, which covers an area of approximately 2 million inhabitants. We quantified the association between sex and survival as well as the effect of covariates such as stage, treatment, and histology in low BMI (BMI < 18.5), normal BMI (BMI, 18.5-25), and high BMI patients (BMI > 25). We showcased the value of the Observational Medical Outcomes Partnership (OMOP) Common Data Model in representing a granular view of all clinical data in a large university hospital setting.^22^ In addition, we used our federated data network to perform external validation of results in four different treatment sites across Europe.

## MATERIALS AND METHODS

### Data Sources, Variables, and Preprocessing

The primary analytical work was performed on the pseudonymized clinical data from Helsinki University Central Hospital (HUS). HUS is responsible for secondary/tertiary cancer care (diagnostics, treatment, and follow-up) of 2.2 million inhabitants. Structured data, including prescribed and administered drugs, medical procedures, laboratory measurements, visits, diagnoses, and patient demographics, were collected from multiple operative medical information systems and harmonized into the OMOP common data model format by mapping local medical codes (eg, drug or procedure codes) to standard OMOP vocabularies and concepts.^22^ In addition, unstructured data (patient notes), including patient journals, admission notes, briefing reports, questionnaires etc, were collected from information systems and used to derive the stage at diagnosis, smoking status, and metastasis status (see the Data Supplement, Appendix S1, for the full description). The full derivation of each clinical feature, preprocessing, and OMOP definitions are provided in the Data Supplement (Appendix S2). This study was conducted in accordance with the EU General Data Protection Regulation, which permits the secondary use of personal health data for scientific research purposes under Article 9(2)(j) and Article 89, and complies with the Finnish Act on the Secondary Use of Health and Social Data (552/2019).

**FIG 1.**
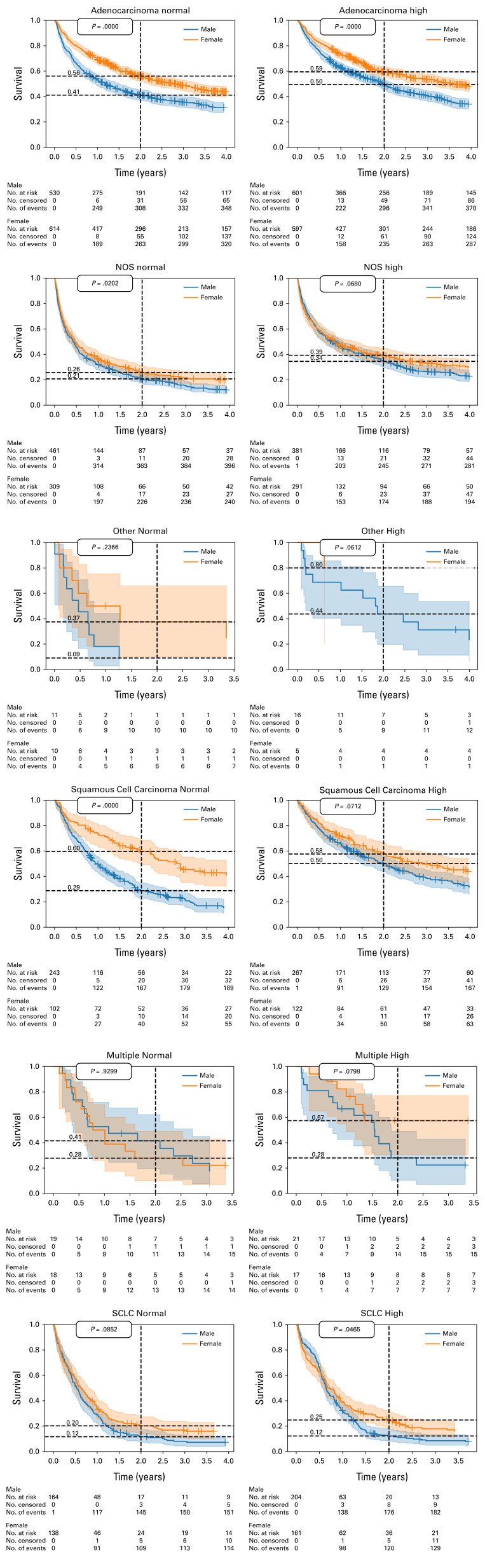
Kaplan-Meier survival curves: sex-related survival differences in normal and high BMI cohorts stratified by histology. *P* values are calculated from a log-rank test. Two-year survivals are visualized by dashed lines.

Four European partner hospitals agreed to perform external federated validation of our results: Antwerp University Hospital (UZA, Belgium), Oslo University Hospital Comprehensive Cancer Centre (Radiumhospitalet, Norway), Copenhagen University Hospital (Rigshospitalet, Denmark), and Leeds Teaching Hospitals NHS Trust (United Kingdom). With these partners, we have established a harmonized OMOP-based data-network that allows rapid real world evidence generation using standardized analytics to optimize testing speed, granularity, and cost-efficiency. The analysis package distributed to the partners comprised two components: a clinical feature comparison between male and female patients within the total, high-BMI, and normal-BMI cohorts, replicating the results shown in Tables 1 and 2; and a univariate survival analysis comparing male and female patients across BMI cohorts, replicating Figure 1. Because of current technical constraints and limitations in the availability of confounder variables at partner sites, multivariate analyses could not be performed.

**TABLE 1.** Patient Characteristics in the Full Patient Group and BMI-Based Cohorts: Low (BMI< 18.5), Normal BMI (BMI, 18.5-25), and High BMI (BMI > 25)

Feature	Value	Full Cohort, No. (%)	Low BMI, No. (%)	Normal BMI, No. (%)	High BMI, No. (%)	Missing BMI, No. (%)
Stage	Stage I	1,160 (15)	43 (11)	430 (16)	426 (16)	261 (15)
	Stage II	125 (2)	7 (2)	44 (1)	49 (2)	25 (1)
	Stage III	1,174 (16)	55 (15)	438 (17)	497 (19)	184 (11)
	Stage IV	2,667 (36)	160 (44)	1,023 (39)	972 (36)	512 (30)
	Unknown	2,201 (30)	95 (26)	685 (26)	741 (28)	680 (41)
Histology	Adenocarc.	2,903 (40)	123 (34)	1,144 (43)	1,198 (45)	438 (26)
	Multiple	96 (1)	6 (2)	37 (1)	38 (1)	15 (1)
	NOS	2,524 (34)	153 (42)	770 (29)	673 (25)	928 (56)
	Other	58 (1)	1 (1)	21 (1)	21 (1)	15 (1)
	SCLC	795 (11)	42 (12)	303 (11)	365 (14)	85 (5)
	Squam.	951 (13)	35 (10)	345 (13)	390 (14)	181 (10)
Smoking	NA	1,478 (20)	92 (25)	447 (17.1)	414 (15)	525 (31)
	Never	217 (3)	3 (1)	79 (3.0)	89 (3)	46 (3)
	Smoker	5,632 (77)	265 (74)	2,094 (80)	2,182 (81)	1,091 (66)
Age, years	59-	851 (12)	29 (8)	321 (12)	387 (14)	114 (7)
	60-69	2,159 (30)	117 (32)	777 (29)	877 (32)	388 (23)
	70-79	3,019 (41)	166 (46)	1,099 (42)	1,121 (41)	633 (38)
	80+	1,298 (18)	48 (13)	423 (16)	300 (11)	527 (31)
First line includes	ICI	365 (5)	14 (4)	168 (6)	163 (6)	20 (1)
	Chemoth.	2,002 (27)	81 (22)	846 (32)	910 (33)	165 (10)
	Surgery	1,143 (16)	33 (9)	474 (18)	629 (23)	7 (1)
	Radioth.	2,690 (37)	118 (33)	861 (33)	852 (31)	859 (51)
	Targeted	308 (4)	12 (3)	115 (4)	112 (4)	69 (4)
Sex	Female	3,354 (45)	198 (55)	1,191 (45)	1,193 (44)	772 (47)
	Male	3,973 (54)	162 (45)	1,429 (54)	1,492 (55)	890 (54)
	Total	7,327	360	2,620	2,685	1,662

Abbreviations: Adenocarc., adenocarcinoma; Chemoth., chemotherapy; ICI, immune checkpoint inhibitor therapy; NA, not available; NOS, nonspecified; Radioth., radiotherapy; SCLC, small cell lung cancer; squam., squamous cell carcinoma.

**TABLE 2.** Comparison of Clinical Covariates Between Male and Female Patients in Normal BMI and High BMI Cohorts

Feature	Value	Normal, No. (%)			High, No. (%)		
		Female Patients	Male Patients	*P*	Female Patients	Male Patients	*P*
Histology	Adenoc.	614 (52)	530 (37)	<.001	597 (50)	601 (40)	<.001
	Multiple	18 (2)	19 (1)	1.000	17 (1)	21 (1)	1.000
	NOS	309 (26)	461 (32)	<.001	291 (24)	382 (26)	.457
	Other	10 (1)	11 (1)	.821	5 ()	16 (1)	.163
	SCLC	138 (12)	165 (12)	1.000	161 (13)	204 (14)	.860
	Squam.	102 (9)	243 (17)	<.001	122 (10)	269 (18)	<.001
Stage	NA	317 (27)	368 (26)	.401	315 (26)	427 (29)	.242
	Stage I	228 (19)	202 (14)	<.001	211 (18)	215 (14)	.020
	Stage II	14 (1)	30 (2)	.120	20 (2)	29 (2)	.657
	Stage III	181 (15)	257 (18)	.083	210 (18)	287 (19)	.357
	Stage IV	451 (38)	572 (40)	.079	437 (37)	535 (36)	.835
Smoking	NA	199 (17)	248 (17)	.705	198 (17)	216 (14)	.270
	Never	66 (6)	13 (1)	<.001	60 (5)	29 (2)	<.001
	Smoker	926 (78)	1,168 (82)	.011	935 (78)	1,248 (84)	.001
First line includes	Chemotherapy	360 (30)	486 (34)	.023	377 (32)	533 (36)	.062
	Radiotherapy	368 (31)	493 (34)	.065	354 (30)	498 (33)	.117
	Surgery	259 (22)	215 (15)	<.001	310 (26)	319 (21)	.004
	ICI	82 (7)	86 (6)	.420	66 (6)	97 (6)	.291
	Targeted	84 (7)	31 (2)	<.001	61 (5)	51 (3)	.058

NOTE. Distributions of categorical features were compared for each category by using a chi-square test, and numerical values by using a Mann-Whitney U test.

Abbreviations: Adenocarc., adenocarcinoma; Chemoth., chemotherapy; ICI, immune checkpoint inhibitor therapy; NA, not available; NOS, nonspecified; Radioth., radiotherapy; SCLC, small cell lung cancer; squam., squamous cell carcinoma.

### Patient Cohorts and Exclusions

First, all patients with lung cancer (International Classification of Diseases codes [ICD]10 C34) diagnosed between January 1, 2015, to December 31, 2024, were selected. Patients with nonmalignant disorders (ICD-10 C34.x7) were excluded from the cohort. Patients who were not registered at HUS at least 1 month before lung cancer diagnosis were further excluded to ensure data availability at the moment of diagnosis, which led to filtering out 478 (6%) of patients (Data Supplement, Fig S1). Patients who had any other cancer diagnosed before lung cancer diagnosis and have received any systemic anticancer treatment 1 year before lung cancer diagnosis (n = 183) were excluded to reduce the variance in clinical outcomes and interpretation of the results, and to simplify automated data processing of unstructured data.

BMI was used to classify patients into cohorts. BMI is defined as patient weight (kg) divided by the square of patient height (m). To derive BMI at diagnosis, we took the average value of all BMI measurements 60 days before diagnosis and maximum 60 days after or until the first treatment. Where BMI was not available, we derived it from independent height and weight measurements if they were accessible. Patients were stratified on the basis of BMI into three subcohorts—low BMI (BMI < 18.5, n = 360), normal BMI (BMI, 18.5-25, n = 2,620), and high BMI (BMI > 25, n = 2,685). To verify that the BMI distribution does not contain significant outliers, we plotted the distribution in the Data Supplement (Fig S2). The distribution is a right-skewed normal distribution, with values up to 50.

### Survival Analysis

Survival was defined as the time from the date of diagnosis to the date of death. Date of diagnosis was defined as the date of the first appearance of lung cancer diagnosis code (any ICD C34.xx excluding nonmalignant codes C34.x7). The follow-up time for all patients in HUS is until September 1, 2025, with at least 8 months of follow-up for each patient. The difference of distribution of clinical variables (histology, age, sex, stage, smoking status, blood measurements, and comorbidity index) between cohorts was compared by using Pearson's chi-square test for categorical variables and Mann-Whitney U test for numerical variables. For the comparison of single covariates between cohorts, we used a z-test of proportions (categorical values) and a Mann-Whitney U test (numerical values). Bonferroni correction was applied to correct for multiple comparisons. The threshold for statistical significance is *P* < .05.

Cox^23^ and restricted mean survival time (RMST)^24^ regression were used for survival analysis. Cox regression, Kaplan-Meier survival curves, and statistical testing were performed using Python package “lifelines” (version 0.30.0).^25^ Proportional hazard assumption was tested by the inspection of Schoenfeld residuals. In univariate analysis to estimate the CI of difference-in-differences analysis, we used the method based on naïve test^26^ using the standard error of the difference-in-differences point estimate (derived from the CI of 2-year survival estimates) and multiplying by 1.96.

The RMST analysis was used to estimate the effect of sex on survival differences.^24,27^ RMST represents the average event-free survival time up to a specified time point (Data Supplement, Fig S3). RMST is defined as the area under the Kaplan-Meier curve up to a defined time point and provides a robust measure that does not rely on the assumption of proportional hazards.^28^ The effect of covariates was evaluated by regression methodology.^29^ R package “survrm2” was used for RMST regression, *P* values, and CIs.

The study adhered to the Declaration of Helsinki and to European and national data privacy regulations. The use of clinical data was approved by the institutional research board (study permit HUS/179/2024).

## RESULTS

### Patient Characteristics

In total, 7,988 patients with malignant lung cancer diagnosed between January 1, 2015, and December 31, 2024, were identified in HUS patient records (Data Supplement, Fig S1). Of those, 7,510 were registered at HUS at least 1 month before lung cancer diagnosis. After filtering out patients with other cancers and a systemic anticancer treatment within 1 year before lung cancer, the full study cohort included 7,327 patients, of whom 51% were male. Patients with previous cancer diagnosis who received systemic anticancer treatment were excluded to reduce the survival variance caused by other cancers, clarify the interpretation of medical records (Data Supplement, Appendix S2), and simplify interpretability of results. Patients were stratified into three subcohorts on the basis of BMI thresholds—low BMI (BMI < 18.5, n = 360), normal BMI (BMI, 18.5-25, n = 2,620) and high BMI (BMI > 25, n = 2,685; Table 1). Among patients with available BMI measurements, high BMI was observed in 47% of males and 48% of females. These proportions are lower than those reported for the general Finnish population in comparable age groups (75% in males and 70% in females).^30^

Adenocarcinoma was the most frequent histology in the full cohort (n = 2,903), followed by not specified (NOS; n = 2,524) and squamous cell carcinoma (n = 951). NOS includes patients for whom the biopsy was not taken, for example, due to frailty and the diagnosis was recorded only on the basis of computed tomography or magnetic resonance imaging scan, or patients in whom histology was not determined from biopsy. Patients in the age group 70-79 years were the largest patient group in all three cohorts, and patients in the age of 80+ years were underrepresented in the high BMI cohort, representing only 11%, with corresponding share in the full cohort being 18% (*P* < .001).

Treatment lines were derived from the data on the basis of the time intervals and treatment types (Methods). Treatment options were classified into surgery, chemotherapy, immune checkpoint inhibitors (ICI), radiotherapy, and targeted therapy. Sankey plot of the first three treatment lines is shown in the Data Supplement (Fig S4). Radiotherapy was the most used therapy in the first-line treatment, administered either alone (n = 2,037) or in combination (n = 580). In the full cohort, 3,157 patients (43%) received only one line of therapy.

### Comparison of Clinical Features Between Male and Female Patients With Normal and High BMI

Sex differences at baseline (time of diagnosis) in clinical features for the patients in normal and high BMI cohorts are summarized in Table 2. In the normal BMI cohort, female patients had lower stage at diagnosis, as 20% of female patients were diagnosed with stage I to II lung cancer, while for male patients, the share was 16% (*P* < .01). Adenocarcinoma was more common among female patients (52% female *v* 38% male, *P* < .001), while squamous cell carcinoma was more common among male patients (9% female *v* 17% male, *P* < .001).

The proportion of never smokers was larger in female patients (6% female *v* 1% male, *P* < .001). Male patients also had a higher Charlson comorbidity index^31^ (Data Supplement, Appendix S1). Female patients received targeted therapy (7% female patients *v* 2% male patients, *P* < .001) and surgical treatment (22% female patients *v* 15% male patients, *P* < .001) more often. Higher surgery rate is likely caused by lower stage at diagnosis (Table 2), while the higher rate of targeted therapies is potentially driven by higher occurrence of targetable mutations among female patients.

### Survival Difference Between Males and Females Is Lower in High BMI Patients in Univariate Analysis, Especially in Squamous Cell Carcinoma

In the full cohort (Fig 1A), survival in females in univariate analysis was significantly longer than in males (*P* < .001), with a 32% 2-year survival rate in males and 43% in females. Among patients within the normal BMI cohort, 2-year survival difference was 15% (44% female and 29% male).

The difference was smaller among patients with high BMI—10% (40% male and 50% female 2-year survival rates, *P* < .001; Fig 1D). The result was also validated by a Cox regression with an interaction variable, indicating interaction between high BMI and male sex (*P* < .01; Data Supplement, Fig S5).

As survival difference between males and females was smaller among high BMI patients compared with normal, we wanted to see whether this holds for all histologies.

**FIG 2.**
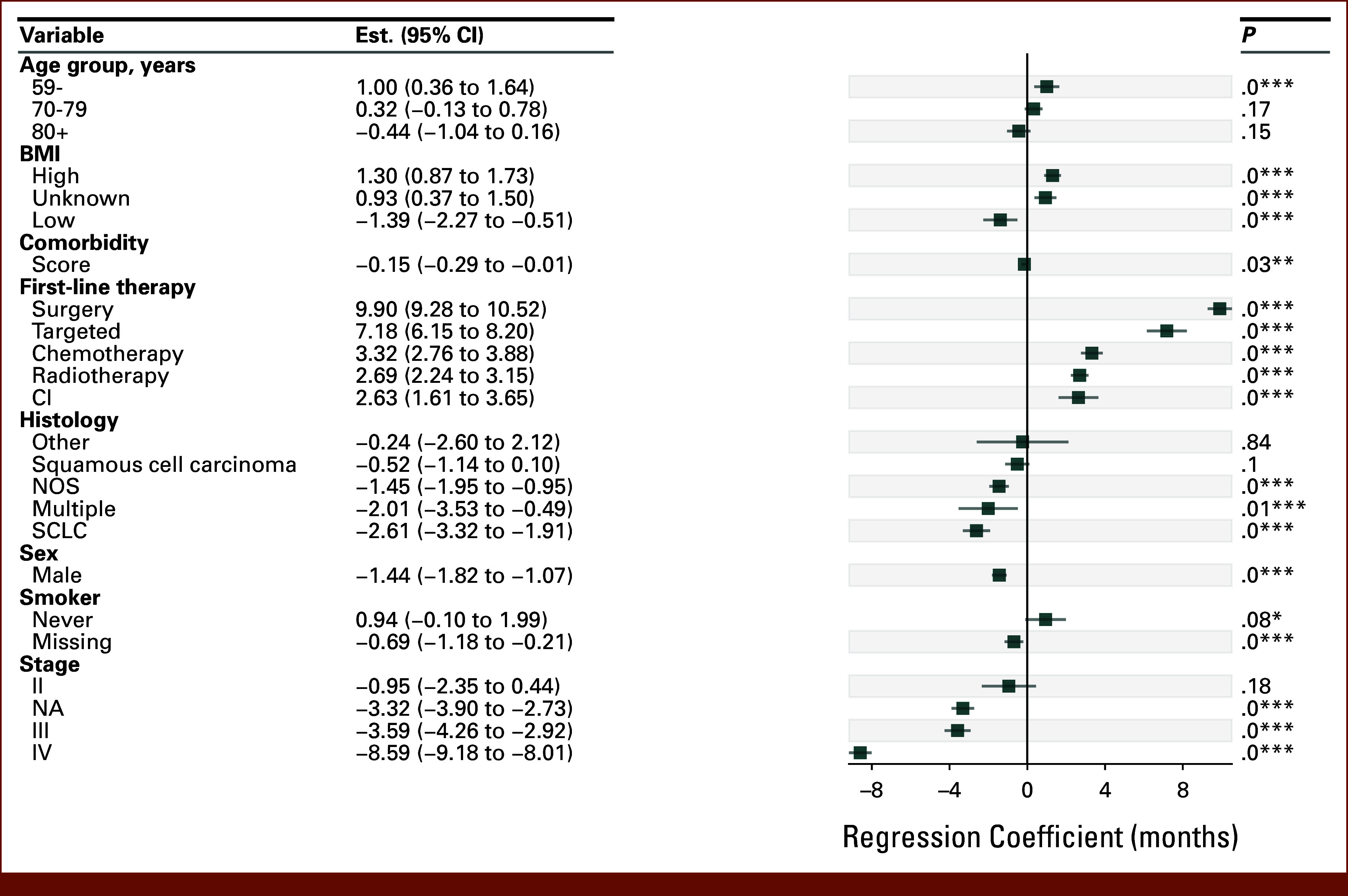
Two-year RMST regression for the full cohort. Baseline categorical covariates are age 60-69 years, normal BMI, no first-line treatment, adenocarcinoma, female sex, smoker, and stage I. *P < .1, **P < .05, ***P < .01. NA, not available; NOS, nonspecified; RMST, restricted mean survival time; SCLC, small cell lung cancer.

We repeated the analysis separately for each histology (Data Supplement, Figs S6-S10). The effect was the largest in squamous cell carcinoma (Fig 2; 2-year survival rates among normal BMI: males—29% females 61%, high BMI: males—51% females 59%). In adenocarcinoma, the effect was moderate (2-year survival rates among normal BMI: males—42% females 59%, high BMI: males—50% females 62%), while in SCLC, there was no effect of high BMI on survival difference.

### External Validation

External validation was conducted in four independent university hospital cohorts with similar patient populations and treatment practices to the HUS patient cohort. For clarity, sites are subsequently referred to by country. Detailed cohort characteristics are provided in the Data Supplement (Tables S1 and S2). Data completeness varied across sites. Denmark exhibited the highest proportion of patients with recorded BMI (98%), whereas Belgium and the United Kingdom had substantially lower BMI data availability (25% and 17%, respectively). Because of insufficient BMI data, the Oslo cohort was excluded from further analyses.

Cohort composition and BMI distribution also differed by site. Only NSCLC cases treated at the department of oncology were available from Denmark. In Belgium, as in Helsinki, adenocarcinoma was the predominant histologic subtype, whereas the UK cohort included a higher proportion of male patients with squamous cell carcinoma. In Helsinki, the proportions of high and normal BMI patients were similar (48%) among both all cases and NSCLC cases. In Denmark, however, there was a higher prevalence of high BMI among males (calculated from the Data Supplement, Fig S10B: 49% high *v* 38% normal) and a lower prevalence among females (38 high *v* 50% normal) compared with Helsinki.

The extent of sex-related differences in survival varied by site (Table 3). The UK cohort demonstrated a difference-in-differences estimate (0.06; 95% CI, –0.03 to 0.16) comparable with Helsinki (0.06; 95% CI, 0.02 to 0.11). In Denmark, the effect size was attenuated (0.02; 95% CI, –0.04 to 0.08), while in Belgium, the effect was minimal and in the opposite direction (–0.01; 95% CI, –0.15 to 0.11), indicating a slightly smaller survival difference between male and female patients with normal BMI compared with those with high BMI. However, the limited sample size and wide CIs in Belgium preclude definitive conclusions.

**TABLE 3.** Summary of 2-Year Point Estimates in Univariate Survival Analysis Among Different Sites

Value	Helsinki	Belgium	Denmark	United Kingdom
High BMI				
Patients	2,524	348	1,647	500
2-year male survival	0.4	0.59	0.48	0.34
2-year female survival	0.51	0.73	0.56	0.35
2-year survival discrepancy	0.11	0.14	0.08	0.01
Normal BMI				
Patients	2,427	363	1,813	380
2-year male survival	0.29	0.53	0.4	0.29
2-year female survival	0.46	0.67	0.5	0.36
2-year survival discrepancy	0.17	0.13	0.1	0.07
Difference-in-differences	0.06	-0.01	0.02	0.06
CI	0.02 to 0.11	–0.15 to 0.11	–0.04 to 0.08	–0.03 to 0.16

NOTE. Difference-in-differences refers to the difference between 2-year survival difference of male and female patients between high and normal BMI cohorts. Overall, it can be concluded that the effect was present in Helsinki stronger than in other locations.

Thus, the observed sex-BMI–related interaction was most pronounced in Helsinki, with similar magnitudes and directions in the UK external validation cohort, with smaller effect observed in other cohorts. These findings underscore the importance of cohort composition and data completeness in the interpretation of sex- and BMI-related survival differences and support our conclusion that BMI should be reported in the upcoming clinical trials and observational studies.

### The Effect of Male Sex on Survival Was 32% Smaller Among High BMI Patients in 2-Year Multivariate Analysis

To understand how covariates affect the effect of sex on survival difference, we performed multivariate regression analyses in high and normal BMI patients. To understand the effects of covariates on all patients before stratified analysis, we ran 2-year RMST regression analyses for the full patient cohort using sex, age, BMI group, histology, smoking status, stage, first-line therapy, and comorbidity index as covariates (Fig 2). As expected, higher stage, age, never smoking, and male sex were associated with lower survival. High BMI was associated with higher survival (RMST, 1.30; ICI, 0.87-1.73), while low BMI was associated with lower survival (RMST, –1.39; ICI, –2.27 to 0.51) than normal BMI, which was used as a baseline cohort. To understand how outliers affect the coefficient of male sex, a dropout outlier analysis was performed and the results are shown in the Data Supplement (Fig S11). We found that the male coefficient varied <2% (from –1.435 to –1.449) during the dropout analysis.

**FIG 3.**
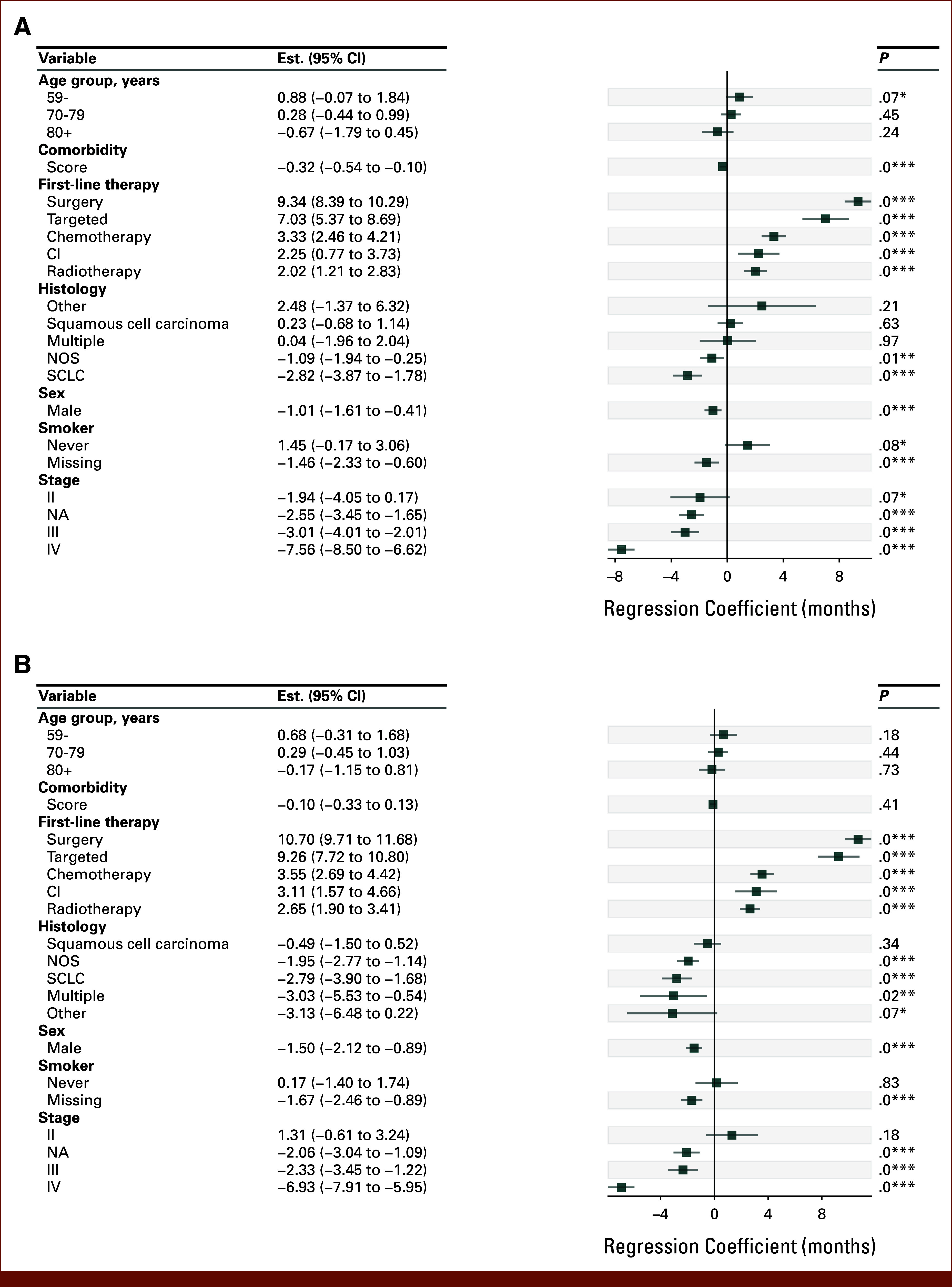
(A) Two-year RMST regression for high BMI patients in the full cohort. Baseline categorical covariates are age 60-69 years, normal BMI, no first-line treatment, adenocarcinoma, female sex, smoker, and stage I. (B) Two-year RMST regression for normal BMI patients in the full cohort. Baseline categorical covariates are age 60-69 years, normal BMI, no first-line treatment, adenocarcinoma, female sex, smoker, and stage I. *P < .1, **P < .05, ***P < .01. NA, not available; NOS, nonspecified; RMST, restricted mean survival time; SCLC, small cell lung cancer.

To understand how BMI influences the effect of sex on survival, multivariate regression was performed separately for high BMI (Fig 3A) and normal BMI (Fig 3B) patients using age, sex, group, histology, smoking status, stage, first-line therapy, and comorbidity index as covariates. The effect of male sex was reduced by 32% in high BMI patients (RMST, –1.01; ICI, –1.61 to –0.41, *P* < .001) compared with normal BMI patients (RMST, –1.50; ICI, –2.12 to –0.89, *P* < .001).

## DISCUSSION

In this study, we demonstrate the clinical utility of large-scale cancer data harmonization^32^ using OMOP methodologies, enabling granular analyses of patient-level factors influencing lung cancer survival. Our findings indicate that higher BMI confers a survival advantage in male patients, with the magnitude of this effect varying by histologic subtype.

We identified that the survival difference between males and females is smaller among patients with higher BMI as high BMI is associated with a larger survival benefit in males than in females. We have also shown the variation of the effect by histology, underlying the need for further research on less frequently occurring histologies such as squamous cell carcinoma. We also confirmed that the rate of patients with high BMI is lower among patients with lung cancer than in the whole Finnish population.

From a clinical perspective, interaction between male sex and high BMI may indicate that there are biological mechanisms related to higher BMI, which are stronger in males. Earlier, it has been stated that female patients have a stronger immune response to lung cancer.^33^ As high BMI is also associated with immune system activity,^34^ it may have an impact on the poorer immune response in male patients. In addition, BMI may act as a proxy for another biological feature. Previous studies^16,35^ have tried to differentiate between patients with high muscle mass and patients with high fat percentage to understand which of them are driving better survival rates.

External validation across cohorts from Belgium, Denmark, and the United Kingdom revealed substantial heterogeneity in data completeness, histologic subtypes, and BMI distributions. Compared with the worldwide^36^ 2:1 ratio in adenocarcinoma and squamous cell carcinoma, in HUS, the rate was 3:1, while in United Kingdom, the rate was 1:1. This aligns with the trend of increasing adenocarcinoma incidence between 2000 and 2019, which has been earlier shown in Finland.^37^

Even more variation was detected in sex. Although in HUS the share of male patients was 54%, in Belgium, it was 58%, but in Denmark, it was as low as 46%. Furthermore, for normal BMI patients in Denmark, the rate was 43%, but for high BMI patients, it was 52%. Overall, this supports our conclusion that BMI should be reported in clinical trials and observational studies as it demonstrates unexpected association with other features and survival. For this study, it implies that the relationship between BMI, sex, and survival gap is complex, and more research work on additional covariates is warranted.

In conclusion, our findings support the inclusion of BMI as a stratification variable in observational lung cancer survival analyses and clinical trial reporting. The observed associations between BMI, sex, and histology warrant further investigation, particularly in less common subtypes such as squamous cell carcinoma. We encourage the inclusion of BMI as a key covariate in clinical trials and observational studies.

## Data Availability

A data sharing statement provided by the authors is available with this article at DOI https://doi.org/10.1200/CCI-25-00263.
